# Structural homology and binding ability comparisons of epitope pair candidates for molecular mimicry triggering of type 1 diabetes mellitus

**DOI:** 10.1093/oxfimm/iqag013

**Published:** 2026-09-02

**Authors:** Ryan Gardner, Joshua Wilkins, Sejal Mistry, Ramkiran Gouripeddi, Julio C Facelli

**Affiliations:** Weber State University, 3848 Harrison Blvd, Ogden, UT 84408, United States; Department of Biomedical Informatics, University of Utah, 421 Wakara, Salt Lake City, UT 84108, United States; Department of Biomedical Informatics, University of Utah, 421 Wakara, Salt Lake City, UT 84108, United States; North Carolina A&T, 1601 E. Market Street, Greensboro, NC 27411, United States; Department of Biomedical Informatics, University of Utah, 421 Wakara, Salt Lake City, UT 84108, United States; Department of Biomedical Informatics, University of Utah, 421 Wakara, Salt Lake City, UT 84108, United States; Utah Clinical and Translatinal Science Institute, University of Utah, 421 Wakara, Salt Lake City, UT 84108, United States; Department of Biomedical Informatics, University of Utah, 421 Wakara, Salt Lake City, UT 84108, United States; Utah Clinical and Translatinal Science Institute, University of Utah, 421 Wakara, Salt Lake City, UT 84108, United States

**Keywords:** Molecular Mimicry, T1DM, Protein Structure Prediction

## Abstract

Molecular mimicry is the process by which foreign peptides contain epitopes similar to self-peptides, potentially inducing autoimmune responses. Identifying potential molecular mimics and studying their properties is key to understanding the onset of autoimmune diseases such as type 1 diabetes mellitus (T1DM). Previous work identified pairs of infectious epitopes (E_INF_) and T1DM epitopes (E_T1D_) that demonstrated sequence homology; however, structural homology and binding ability to HLA molecules were not considered. Correlating sequence homology with structural properties and binding ability is important for translational investigation of potential molecular mimics. This work compares sequence homology with structural homology and binding ability by calculating the structures and binding ability of the epitope pairs identified in previous work. For each pair of E_INF_ and E_T1D_ we calculated the root mean square deviations (RMSD) between their predicted isolated structures and their percent (%) overlap when bound to the corresponding HLA molecule using the Boltz-2. Of the 53 epitope pairs considered here, only 6 were found to not exhibit any matching (defined as less than 3 residues overlap). For the other epitopes, the RMSD ranges from 0.13 Å to 1.39 Å with an average of 0.56 Å. The calculated % overlaps between the infectious and T1DM epitopes when bound to the corresponding HLA molecules ranges from 0% to 93.3%. There are 3 pairs that show nearly identical HLA footprints, 15 very similar binding mode, 19 similar binding mode, 11 moderately similar, and only 5 showing a totally different footprint. Most of the E_INF_/E_T1D_ pairs selected by sequence homology show similar structural and binding ability to HLA molecules, indications that may lead to disease onset due to molecular mimicry. These findings suggest that searching for epitope pairs using sequence homology, a much less computationally demanding approach, leads to plausible candidates for molecular mimicry that should be considered for further experimental validation. However, full binding ability calculations at scale may be necessary to advance the *in-silico* molecular mimicry predictions, which may be useful to select the most promising candidates for experimental studies.

## Background

Molecular mimicry happens when external and self-peptides containing similar epitopes trigger a cross-reactive immune response [1–6]. In susceptible individuals, molecular mimicry has been postulated as a mechanism for the onset of autoimmune diseases, including type 1 diabetes mellitus (T1DM) [7–9]. T1DM is a chronic condition characterized by the patient’s inability to produce insulin due to an autoimmune insult. Insulin is a hormone produced in pancreatic β-cells that regulates carbohydrate metabolism. Understanding molecular mimicry can lead to the understanding of T1DM etiology and reveal novel paths for disease prevention and drug design.

The global incidence of T1DM has increased in recent years, but its cause is largely unknown; however, it is thought that viral infections could be a significant contributor to this trend [10, 11]. It is difficult to associate this steady rise of the incidence of T1DM with a single factor, such as genetics, given the heterogeneity of the condition [12], but it is likely the result of the complex interplay of multiple factors (i.e. genetic, hygiene, dietary, environmental, viral), with molecular mimicry having an important role in this problem.

Previously, Mistry *et al*. [13] reported a pipeline for investigating molecular mimicry by finding homologous peptides implicated in T1DM etiology and infectious agents. This pipeline generates a prioritized list of infectious epitopes (E_INF_) showing sequence homology with T1DM epitopes (E_T1D_), with similar empirical binding affinity to specific major histocompatibility complexes (MHC), and similar empirical potential for T-cell immunogenicity. But the previous study did not consider structural homology of the epitope pairs (E_INF_/E_T1D_ pairs) found by sequence homology or binding ability. While sequence homology may provide insight into potential molecular mimics at a much lower computational cost, it is necessary to compare structural and binding characteristics to the HLA molecules as, ultimately, molecular mimicry will depend on the ability of the E_INF_ peptide–MHC presentation and T-cell receptor recognition. This second line of investigation is necessary to produce more promising candidates. More importantly, while structural similarity of the isolated T1DM and infectious epitopes may be important, ultimately the peptide conformation may be influenced by MHC binding and TCR-facing residue orientation, nesting models that take into consideration the interaction between the epitopes and the HLA molecules.

Recent computational advancements make possible the prediction of polypeptide structures and binding affinities. As shown in the results presented at a recent conference [14], it is now possible to use state of the art 3D protein structure prediction methods to study structural homology for relative large sets of epitope pairs. Moreover, in recent years there has been an increased interest in using *in-silico* methods to study antibody epitope interactions and their effect on immunity. For instance, Balbin *et al*. introduced Epitopedia, [5, 6] a comprehensive framework for studying molecular mimicry. Desta *et al*. [15] used in silico methods including structure prediction with Alphafold [16, 17] and PIPER docking [18] to model antibody-antigen interactions. Maleki *et al*. [19] used a comprehensive set of *in-silico* tools to design a recombinant multi-epitope vaccine against influenza A, while Russo *et al*. used similar methods to design a COVID-19 vaccine [18]. Recent advances in AI based open-source models, like Boltz-2 [20, 21], have also shown the ability to reliably predict binding similarities between proteins and small peptides and molecules.

Here we present a comprehensive structural study by comparing the predicted structures and binding abilities to HLA molecules, calculated with Boltz-2, of all the E_INF_/E_T1D_ pairs reported by Mistry *et al*. [13] to evaluates whether sequence-derived candidates also exhibit structural similarity and comparable HLA-binding footprints would better emphasize the novelty and significance of the study.

## Results and discussion

Among all the cases analyzed here, model_0 from the Boltz-2 predictions consistently exhibited the highest confidence scores. For these models, the confidence scores show an average of 0.86, with a standard deviation of 0.02, a Max of 0.90, and a Min of 0.77. The scores for all models considered here are presented in the Supplementary Material Table S1, available as supplementary material at *OXFIMM* online. The great majority of these scores are considered very good [20, 21], indicating that the results discussed here for all model structures are reliable computational predictions of the isolated structures of the epitopes and their binding regions to HLA molecules.

Table 1 presents the RMSD and % Overlap between E_INF_/E_T1D_ pairs. The RMSD corresponds to the comparison of the isolated structures, and % Overlap refers to the calculated similarity when bound to their corresponding HLA molecules. Pair definitions and amino acid sequences of both the T1DM and infectious epitopes can be found in the supplementary material Table S2, available as supplementary material at *OXFIMM* online. The Figs depicting the structural overlap of all E_INF_/E_T1D_ pairs considered here when bound to their respective HLA molecules are depicted in the supplementary material Figure S1, available as supplementary material at *OXFIMM* online.

**Table 1:** RMSD and % Overlap between EINF/ET1D pairs.

Pair	Overlap (%)	Rank	RMSD
1	65	41	0.31
2	46	49	no match
3	75	28	0.34
4	58	46	0.22
5	87	5	0.45
6	70	36	0.73
7	78	24	no match
8	85	9	0.25
9	81	17	no match
10	73	34	0.17
11	7	52	0.65
12	66	40	0.47
13	81	18	no match
14	83	14	0.85
15	78	23	0.14
16	63	43	0.62
17	14	50	no match
18	11	51	0.87
19	56	48	0.47
20	0	53	no match
21	65	42	0.47
22	74	32	0.48
23	70	37	0.32
24	77	26	0.3
25	59	45	0.52
26	58	47	0.55
27	74.3	30	0.44
28	78.1	21	0.5
29	82.4	15	0.72
30	87.5	3	0.56
31	90.3	2	0.53
32	86.2	7	0.67
33	86.7	6	0.75
34	75.8	27	1.1
35	62.9	44	1.13
36	78.1	22	0.13
37	69.7	38	1.25
38	75	29	0.2
39	83.9	12	0.41
40	87.5	4	1.24
41	69.7	39	1.38
42	84.4	10	0.16
43	74.1	31	0.38
44	79.3	19	0.99
45	93.3	1	0.19
46	73.5	33	1.39
47	85.3	8	0.59
48	73	35	0.49
49	84.4	11	0.24
50	77.4	25	0.3
51	82.4	16	0.18
52	83.3	13	0.98
53	78.8	20	0.4

RMSD corresponds to the isolated structures and % Overlap refers to the calculated similarity when bound to their corresponding HLA molecules. Rank refers to the rank of the overlap scores, with # 1 being the largest overlap in the set. Pair definitions can be found in the supplementary material Table S2, available as supplementary material at *OXFIMM* online.

Of the 53 epitope pairs considered here, only six that do not exhibit any matching (defined as less than 3 residues overlap) for the isolated structures. For the other epitopes, the RMSD ranges from 0.13 Å to 1.39 Å with an average of 0.56 Å. No significant differences were observed by epitopes binding to different HLA molecules. The calculated overlap between the infectious and T1DM epitopes when bound to the corresponding HLA ranges from 0% to 93.3%. According to the definitions in the methods sections, there are 3 pairs that show nearly identical HLA footprint, 15 very similar binding mode, 19 similar binding mode, 11 moderately similar, and only 5 showing a totally different footprint.

**Figure 1:**
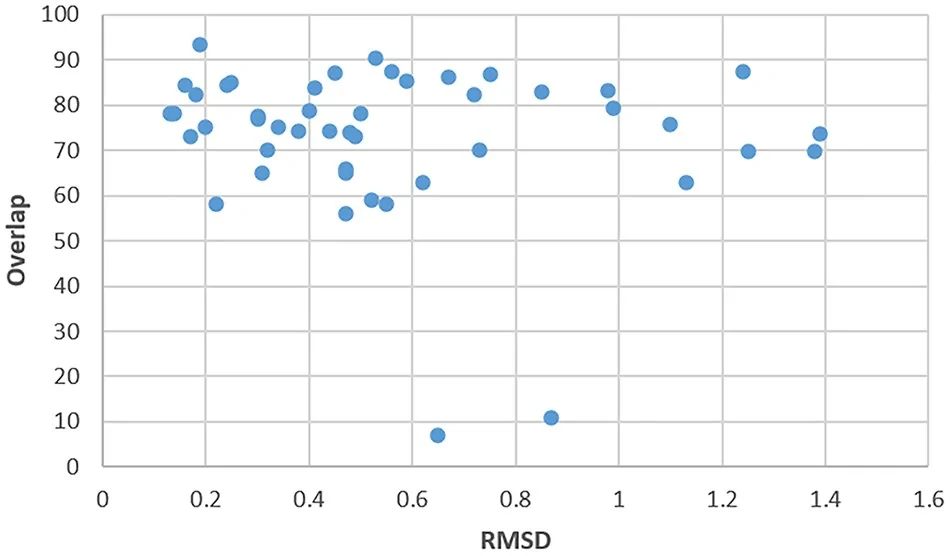
Correlation between the calculated overlap of the epitope pairs when bound to the corresponding HLA molecule versus the RMSD calculated between the predicted structures of the isolated epitopes.

When considering the binding overlapping to the HLA molecules of the six nonstructural matching isolated epitopes, it was observed that three show similar or very similar overlap, one similar, and two different. The lack of correlation between structural similarity of the isolated epitopes and their calculated binding similarities as measured by the overlap does not show any correlation as depicted in Fig. 1. This a strong argument to consider the binding similarity as a much stronger indicator of potential molecular mimicry, than structural similarity of the isolated epitopes.

There are nine different antigens associated with the E_T1D_ studied here. Insulin shows five merged peptide domains, four of which are associated with HLA-A*02:01 and one associated with HLA-B*08:01. GAD65 and IA-2 both contain three merged peptide domains associated with HLA-DRB4*01:01 or HLA-A*02:01. The remaining merged peptide domains were associated with HLA-A*02:01, with ZnT8 containing three merged peptide domains and S100B, KCNK16, PC2, ISL-1, and UCN3 all containing one merged peptide domain. Tables 2–10 show the epitope pairs with overlaps larger than 50%, which are plausible candidates for triggering molecular mimicry.

**Table 2:** Infectious epitopes matching those T1DM epitopes from Glutamate Decarboxylase 2 with overlaps over 50%.

Pair	E-T1D	E-INF	Overlap (%)	E-INF organism
21	KVNFFRMVISNP	NVNILVKQISTP	65.0	Human respiratory syncytial virus A
43	RLLPLLALL	RLNELLAYV	74.1	Human herpesvirus 1
53	ALLGIPLNV	ALLGLTLGV	78.8	Thermococcus onnurineus

**Table 3:** Infectious epitopes matching those T1DM epitopes from Insulin with overlaps over 50%.

Pair	E-T1D	E-INF	Overlap (%)	E-INF Organism
3	LYHVYEV	LYVLFEV	75.0	Human adenovirus C
4	SHLVEAL	ADLVYAL	58.0	Severe acute respiratory syndrome coronavirus 2
6	LTAVAE	LTLFAE	70.0	*Leishmania panamensis*
12	GYIVTDQKPLS	GYIPLVGAPLG	66.0	Hepatitis C virus
13	LLIDLTSFL	LLYDANYFL	81.0	Severe acute respiratory syndrome coronavirus 2
22	YLYNI	YLYNT	74.0	Middle East respiratory syndrome-related coronavirus
23	ALWGPDPAA	ALWALPHAA	70.0	Varicella-zoster virus
24	PDPAAA	PDTAAV	77.0	Hantaan virus
25	VDGVLSV	VDGVPFV	59.0	Middle East respiratory syndrome-related coronavirus
26	DVFHQY	DVFHLY	58.0	Severe acute respiratory syndrome coronavirus 2
27	PLLALL	HLLVLL	74.3	*Leishmania panamensis*
29	LLALLA	LLTLLA	82.4	Dengue virus type 2
30	LLALLAL	LLEWLAM	87.5	Severe acute respiratory syndrome coronavirus 2
31	LLALLAL	LLVTLAI	90.3	Severe acute respiratory syndrome coronavirus 2
32	LLALLAL	LLSTAAL	86.2	Leishmania donovani
33	MLMPVH	KLMPVC	86.7	Severe acute respiratory syndrome coronavirus 2
34	LLPLLA	LLTLLA	75.8	Dengue virus type 2
41	LTAVAEEV	LGAVQNEV	69.7	Hepatitis C virus
42	PRLIAFTSEHSHFS	ARLLSIRAMSTKFS	84.4	*Mycobacterium tuberculosis*
45	AVDGVLS	AVNGVMW	93.3	Hepatitis C virus
46	LLPLLALL	LLVTLAIL	73.5	Severe acute respiratory syndrome coronavirus 2
47	LLPLLALL	LLCLIFLL	85.3	Hepatitis B virus
48	HLVEALYLV	HLDDVGFLV	73	*Mycobacterium tuberculosis*
49	ILKDFSILL	ILSPFLPLL	84.4	Hepatitis B virus
51	RLLPLL	PFLPLL	82.4	Hepatitis B virus

**Table 4:** Infectious epitopes matching those T1DM epitopes from Insulin Gene Enhancer Protein ISL-1 with overlaps over 50%.

Pair	E-T1D	E-INF	Overlap (%)	E-INF Organism
35	LLPLLAL	LLEWLAM	62.9	Severe acute respiratory syndrome coronavirus 2
44	TNHFL	ANHFL	79.3	Mumps orthorubulavirus

**Table 5:** Infectious epitopes matching those T1DM epitopes from Neuroendocrine Convertase 2 with overlaps over 50%.

Pair	E-T1D	E-INF	Overlap (%)	E-INF Organism
50	RLLPLL	HLLVLL	77.4	*Leishmania panamensis*

**Table 6:** Infectious epitopes matching those T1DM epitopes from Potassium Channel Subfamily K Member 16 with overlaps over 50%.

Pair	E-T1D	E-INF		Overlap (%)	E-INF Organism
5	LTAVA	WTAVA	87		*Leishmania donovani*

**Table 7:** Infectious epitopes matching those T1DM epitopes from Protein S100-B with overlaps over 50%.

Pair	E-T1D	E-INF	Overlap (%)	E-INF Organism
1	ALIDV	ALYDV	65	Hepatitis C virus
2	ALIDVFH	ALSGVFC	46	Severe acute respiratory syndrome coronavirus 2
10	HLVEAL	DLVYAL	73	Severe acute respiratory syndrome coronavirus 2

**Table 8:** Infectious epitopes matching those T1DM epitopes from Receptor-type tyrosine-protein phosphatase-like N with overlaps over 50%.

Pair	E-T1D	E-INF	Overlap (%)	E-INF Organism Name
11	IVTDQK	IVTDHR	7	*Mycobacterium tuberculosis*
16	AVDGVLS	AVLEVLS	63	*Leishmania donovani*
17	IVTDQKPLSLA	ISFDQMERYLA	14	*Leishmania panamensis*
18	IVTDQKPLSLAA	ISFDQMERYLAA	11	*Leishmania panamensis*
19	IVTDQKPLSLAA	IVVNASASEAAA	56	*Prochlorococcus marinus*
20	IVTDQKPLSLAA	IVSTAAQTFLAT	0	Hepatitis C virus
36	TRREAE	TRVEAQ	78.1	Hepatitis C virus
37	RREAE	RRLAE	69.7	Human herpesvirus 6A
38	LALLAL	LAAYAL	75	*Trypanosoma cruzi*
39	ALLAL	ALLAF	83.9	Varicella-zoster virus

**Table 9:** Infectious epitopes matching those T1DM epitopes from Urocortin-3 with overlaps over 50%.

Pair	E-T1D	E-INF	Overlap (%)	E-INF Organism Name
40	ALLAL	ALWAL	87.5	Varicella-zoster virus

**Table 10:** Infectious epitopes matching those T1DM epitopes from Zinc Transporter 8 with overlaps over 50%.

Pair	E-T1D	E-INF	Overlap (%)	E-INF Organism Name
7	LQANPVE	LQFIPVE	78	Hepatitis C virus
8	LLPLLALL	WLPTGTLL	85	Severe acute respiratory syndrome coronavirus 2
9	QANPVEV	DPNPPEV	81	Brassica napus
14	ILAVDGVLSV	FLPVDFFPSV	83	Hepatitis B virus
15	AVDGVLS	AVNGVLW	78	Hepatitis C virus
28	LLALL	LLGLL	78.1	*Mycobacterium tuberculosis*
52	LLPLL	LLGLL	83.3	*Mycobacterium tuberculosis*

In total, the authors have identified 48 infectious epitopes with overlaps larger than 50% that are plausible candidates for molecular mimicry. From those 48 epitopes three are associated with Glutamate Decarboxylase 2, 25 with Insulin, two with Insulin Gene Enhancer Protein ISL-1, one with Neuroendocrine Convertase 2, one with Potassium Channel Subfamily K Member 16, three with Protein S100-B, 10 with Receptor-type tyrosine-protein phosphatase-like N, one with Urocortin-3 and seven with Zinc Transporter 8.

The infectious epitopes in the pairs considered here were derived from 19 pathogens, with the number of epitopes associated with each pathogen shown in parentheses: *Brassica napus* (1); Dengue virus type 2 (2); Hantaan virus (1); Hepatitis B virus (4); Hepatitis C virus (7); Human adenovirus C (1); Human herpesvirus 1 (1); Human herpesvirus 6A (1); Human respiratory syncytial virus A (1); *Leishmania donovani* (3); *Leishmania panamensis* (3); Middle East respiratory syndrome-related coronavirus (2); Mumps orthorubulavirus (1); *Mycobacterium tuberculosis* (4); *Prochlorococcus marinus* (1); Severe acute respiratory syndrome coronavirus 2 (10); *Thermococcus onnurineus* (1); *Trypanosoma cruzi* (1); and Varicella-zoster virus (3). It is quite notable that epitopes from proteins of Severe acute respiratory syndrome coronavirus 2 are present in 10 E_INF/_E_T1D_ epitope pairs that have high-priority for experimental studies of their potential to develop molecular mimicry. This is consistent with recent clinical observations that highlight the potential increase of T1DM cases after the COVID-19 pandemic [22], a topic that requires more study as the computational findings while interesting, do not demonstrate epidemiological association, causal relationship, or functional T-cell cross-reactivity.

## Methods

Previous work [13] identified, catalogued, and indexed 53 unique pairs of infectious epitopes (E_INF_) and T1DM epitopes (E_T1D_) that demonstrated sequence homology and potential for molecular mimicry. Here, the authors predicted the isolated structure of these epitopes using Boltz-2 [20, 21], with default parameters. Boltz-2 is a new machine learning approach that incorporates previous knowledge about protein structure and that leveraging multi-sequence alignments into the design of a deep learning algorithm can predict with great accuracy protein structures and binding ability. While other similar approaches exist, we prefer Boltz-2 over other similar methods because (i) Boltz-2 exhibits similar performance, and (ii) it is an open-source alternative to proprietary options.

The amino acid sequences of the HLA molecules were obtained from (https://www.ebi.ac.uk/ipd/imgt/hla/) and depicted in Table S3, available as supplementary material at *OXFIMM* online. The corresponding antigen epitopes were obtained from IEDB [23] (https://www.iedb.org/) and entered in Table S2, available as supplementary material at *OXFIMM* online. Because Boltz-2 requires a sequence of at least 10 amino acids and many of the epitopes did not meet this requirement, we expanded the epitopes using the full protein sequences from UniProt (https://www.uniprot.org/). Both the E_INF_ and E_T1D_ sequences were expanded symmetrically at both ends to reach the minimum length required by Boltz-2.

The expansion of the epitopes to the minimum required length is a limitation in our approach, but it should be considered as an approximation necessary when using any state-of-the-art 3D protein structure prediction method [20]. While in absence of experimental evidence it is difficult to ascertain the exact implications of this approximation, we compared the structures of the best overlapping epitopes, pair 45, *WMRLLPLLAL* and ***LDHLLVLLEK*** with those extended even more by adding three additional amino acids at each end, *MALWMRLLPLLALLAL* and *SKTLDHLLVLLEKATI*. The Boltz-2 predicted structures of these isolated four amino acid fragments show no differences in the central regions corresponding to the original ones, even more important when the epitopes are bound to the corresponding HLA molecules the four models show that the superposition, Figure S2, available as supplementary material at *OXFIMM* online, is almost perfect in the original epitopes binding region, supporting the use of expansion of the epitopes as done here and arguing that end effects may not affect the results presented here. Of course, due to the limited number of tests, we recognize that this may not be generalizable deserving further studies.

Structures of the isolated epitopes were compared (i.e. superimposed and visualized) with UCSF ChimeraX [24–26] using default parameters. The RMSD was calculated for each E_INF_/E_T1D_ pair of isolated structures utilizing the “matchmaker” tool in ChimeraX with standard parameters.

**Figure 2:**
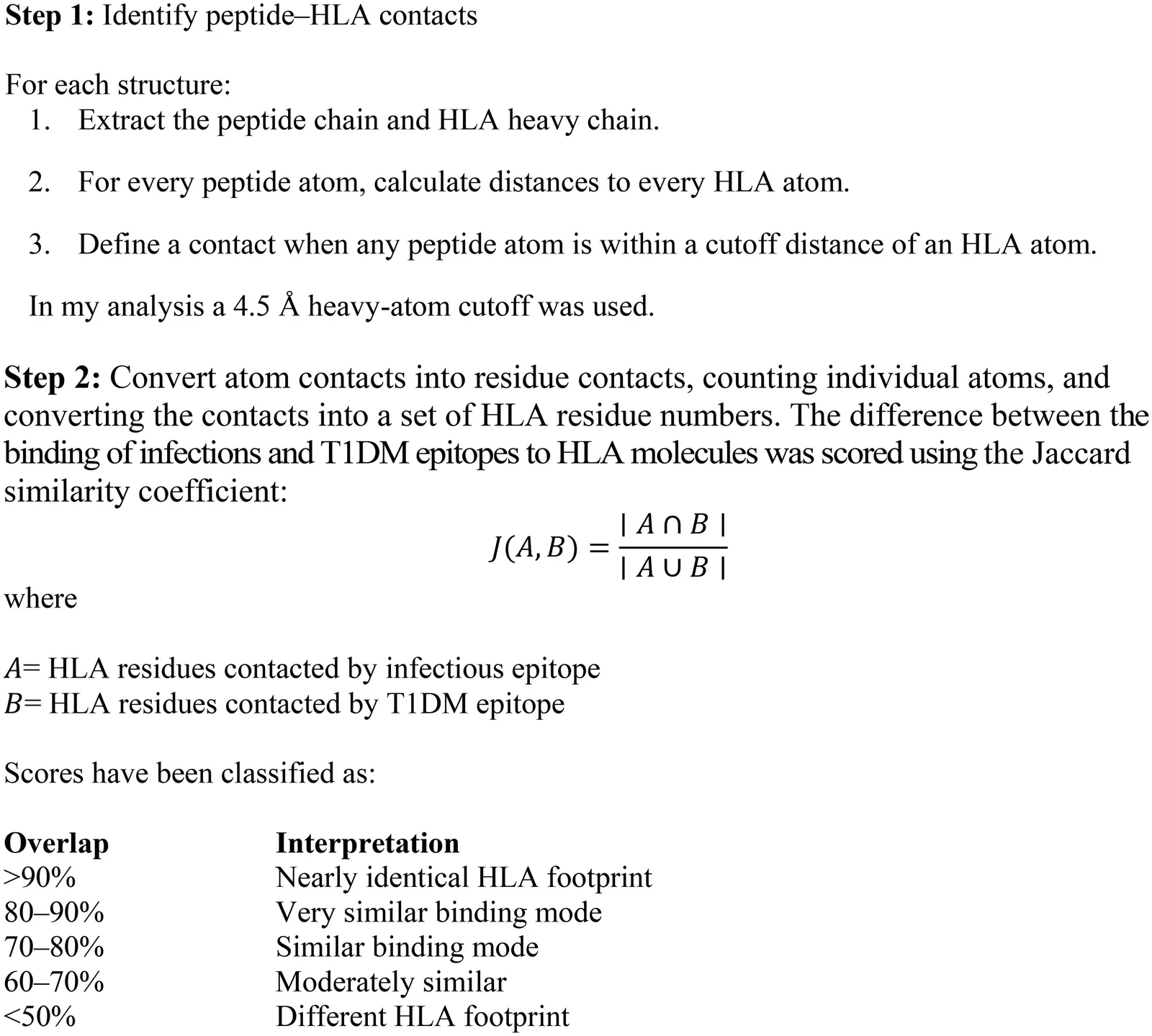
Algorithm implemented in ChatGPT 5.2 for comparing binding infections and T1DM epitopes to HLA molecules.

Binding comparisons were done using the .cif files from the Boltz-2 calculations for the complexes of each individual epitope in the pair with the corresponding HLA molecule. The ability of the infectious fragments to bind at the same groove as the T1DM epitope peptide was quantified by the % Overlap and classified as “Strong,” “Partial,” and “No overlap” using the following algorithm (Fig. 1) implemented in the University of Utah instance of ChatGPT 5.2 on 20 April 2026. As indicated in step 2 of the algorithm, these categories are somehow arbitrary and are used as categorical variables derived from ranges of the percentages overlap calculated with the Jaccard similarity coefficient (Fig. 2).

All results reported here were further verified by one of the authors (JCF), and none of the results were directly imported from ChatGPT without human supervision.

Full results of the Boltz-2 calculation are in files Epitope_Structures.zip for the isolated epitopes and Pairs_Structures.zip for the complex of the epitopes with the corresponding HLA molecules in the ZENODO repository: 10.5281/zenodo.20600080.

## Conclusions

The results presented here show that the Mistry *et al*.’s sequence homology pipeline [13] is successful for finding epitope pairs that also show structural homology and similar binding characteristics to HLA molecules. Overall, close to 90% of the epitopes identified by sequence homology show binding similarities of 50% or larger, making them good candidates for triggering molecular mimicry and making them targets for further investigation. The results also provide evidence that sequence and structural homology of the isolated epitopes are not the whole picture; the calculations presented here can find epitope-HLA candidate pairs with similar peptide presentation within the HLA groove that may increase their likelihood of T-cell cross-recognition. The results presented here using recent advances in protein binding using methods like Boltz-2 show that it is possible to develop pipelines to predict epitope-HLA complexes for hundreds of epitope pairs, but likely sequence homology will be necessary for very large scale scans like the one recently reported by Maldonado *et al*. [27].

There are some important limitations from this study. While the computational findings are interesting, the present work does not demonstrate epidemiological association, causal relationship, or functional T-cell cross-reactivity, which will require clinical and/or experimental studies. Finally, while this study shows that the methods based on sequence homology show a relatively low false prediction rate (less than 10% in this study), the study does not provide any information about missing epitopes that could exhibit strong binding affinity without sequence homology.

## Supplementary Material

iqag013_Supplementary_Data

## Data Availability

The data presented in this study are openly available in ZENODO repository: https://doi.org/10.5281/zenodo.20600080.
