# Supplementary material for: Structural homology and binding ability comparisons of epitope pair candidates for molecular mimicry triggering of type 1 diabetes mellitus
Source: Oxf Open Immunol. 2026 Sep 2;7(1):iqag013. doi: 10.1093/oxfimm/iqag013 (PMC13602411; doi:10.1093/oxfimm/iqag013)
Supplement: Supplementary file 1 [file iqag013_supplementary_data.docx]

**Supplementary Materials**

**Table S1**: Confidence scores of all Boltz-2 model_0 structures considered here. Pair numbering is given in Supplementary Materia Table S2.

| **Pair** | **Pair #** | **Confidence Score** |
| --- | --- | --- |
|  |  |  |
| Pair_E-INF | 1 | 0.90 |
| Pair_E-INF | 2 | 0.86 |
| Pair_E-INF | 3 | 0.89 |
| Pair_E-INF | 4 | 0.85 |
| Pair_E-INF | 5 | 0.89 |
| Pair_E-INF | 6 | 0.84 |
| Pair_E-INF | 7 | 0.87 |
| Pair_E-INF | 8 | 0.89 |
| Pair_E-INF | 9 | 0.87 |
| Pair_E-INF | 10 | 0.87 |
| Pair_E-INF | 11 | 0.84 |
| Pair_E-INF | 12 | 0.84 |
| Pair_E-INF | 13 | 0.88 |
| Pair_E-INF | 14 | 0.87 |
| Pair_E-INF | 15 | 0.88 |
| Pair_E-INF | 16 | 0.83 |
| Pair_E-INF | 17 | 0.80 |
| Pair_E-INF | 18 | 0.84 |
| Pair_E-INF | 19 | 0.85 |
| Pair_E-INF | 20 | 0.80 |
| Pair_E-INF | 21 | 0.80 |
| Pair_E-INF | 22 | 0.88 |
| Pair_E-INF | 23 | 0.88 |
| Pair_E-INF | 24 | 0.85 |
| Pair_E-INF | 25 | 0.88 |
| Pair_E-INF | 26 | 0.86 |
| Pair_E-INF | 27 | 0.85 |
| Pair_E-INF | 28 | 0.86 |
| Pair_E-INF | 29 | 0.88 |
| Pair_E-INF | 30 | 0.87 |
| Pair_E-INF | 31 | 0.89 |
| Pair_E-INF | 32 | 0.86 |
| Pair_E-INF | 33 | 0.87 |
| Pair_E-INF | 34 | 0.88 |
| Pair_E-INF | 35 | 0.86 |
| Pair_E-INF | 36 | 0.86 |
| Pair_E-INF | 37 | 0.86 |
| Pair_E-INF | 38 | 0.88 |
| Pair_E-INF | 39 | 0.86 |
| Pair_E-INF | 40 | 0.88 |
| Pair_E-INF | 41 | 0.88 |
| Pair_E-INF | 42 | 0.88 |
| Pair_E-INF | 43 | 0.77 |
| Pair_E-INF | 44 | 0.88 |
| Pair_E-INF | 45 | 0.85 |
| Pair_E-INF | 46 | 0.89 |
| Pair_E-INF | 47 | 0.87 |
| Pair_E-INF | 48 | 0.86 |
| Pair_E-INF | 49 | 0.85 |
| Pair_E-INF | 50 | 0.89 |
| Pair_E-INF | 51 | 0.89 |
| Pair_E-INF | 52 | 0.84 |
| Pair_E-INF | 53 | 0.86 |
| Pair_E-T1D | 1 | 0.88 |
| Pair_E-T1D | 2 | 0.89 |
| Pair_E-T1D | 3 | 0.86 |
| Pair_E-T1D | 4 | 0.86 |
| Pair_E-T1D | 5 | 0.87 |
| Pair_E-T1D | 6 | 0.86 |
| Pair_E-T1D | 7 | 0.87 |
| Pair_E-T1D | 8 | 0.87 |
| Pair_E-T1D | 9 | 0.88 |
| Pair_E-T1D | 10 | 0.89 |
| Pair_E-T1D | 11 | 0.82 |
| Pair_E-T1D | 12 | 0.90 |
| Pair_E-T1D | 13 | 0.87 |
| Pair_E-T1D | 14 | 0.85 |
| Pair_E-T1D | 15 | 0.87 |
| Pair_E-T1D | 16 | 0.87 |
| Pair_E-T1D | 17 | 0.83 |
| Pair_E-T1D | 18 | 0.84 |
| Pair_E-T1D | 19 | 0.84 |
| Pair_E-T1D | 20 | 0.84 |
| Pair_E-T1D | 21 | 0.79 |
| Pair_E-T1D | 22 | 0.87 |
| Pair_E-T1D | 23 | 0.87 |
| Pair_E-T1D | 24 | 0.84 |
| Pair_E-T1D | 25 | 0.87 |
| Pair_E-T1D | 26 | 0.85 |
| Pair_E-T1D | 27 | 0.86 |
| Pair_E-T1D | 28 | 0.86 |
| Pair_E-T1D | 29 | 0.86 |
| Pair_E-T1D | 30 | 0.87 |
| Pair_E-T1D | 31 | 0.86 |
| Pair_E-T1D | 32 | 0.88 |
| Pair_E-T1D | 33 | 0.88 |
| Pair_E-T1D | 34 | 0.86 |
| Pair_E-T1D | 35 | 0.85 |
| Pair_E-T1D | 36 | 0.86 |
| Pair_E-T1D | 37 | 0.86 |
| Pair_E-T1D | 38 | 0.89 |
| Pair_E-T1D | 39 | 0.84 |
| Pair_E-T1D | 40 | 0.88 |
| Pair_E-T1D | 41 | 0.88 |
| Pair_E-T1D | 42 | 0.87 |
| Pair_E-T1D | 43 | 0.81 |
| Pair_E-T1D | 44 | 0.87 |
| Pair_E-T1D | 45 | 0.86 |
| Pair_E-T1D | 46 | 0.88 |
| Pair_E-T1D | 47 | 0.88 |
| Pair_E-T1D | 48 | 0.88 |
| Pair_E-T1D | 49 | 0.89 |
| Pair_E-T1D | 50 | 0.86 |
| Pair_E-T1D | 51 | 0.88 |
| Pair_E-T1D | 52 | 0.86 |
| Pair_E-T1D | 53 | 0.88 |

**Table S2: C**omplete results of the work presented here. Amino acid letters in RED correspond to the original sequences of the epitopes, while those in back are those added to satisfy the minimum length needed for Boltz-2 predictions. Structures of the isolated epitopes were compared, visualized with UCSF ChimeraX using default parameters. The RMSD was calculated for each E_INF_/E_T1D_ pair by utilizing the “matchmaker” tool in ChimeraX. Binding comparisons were done using the .cif files from the Boltz-2 calculations for the complexes of each individual epitope in the pair with the corresponding HLA molecule. The ability of the epitope to bind at the same groove as the T1DM epitope peptide was classified as Strong, Partial and No overlap using an algorithm (Fig. 1) implemented in the University of Utah instance of ChatGPT 5.2 on April 20th, 2026. Rank order is the rank of the overlap scores, with # 1 being the largest overlap in the set.

| **Pair** | **E-T1D** | **E-T1D (EXT)** | **E-INF** | **E-INF (EXT)** | **Overlap (%)** | **Rank** | **RMSD** | **E-T1D MHC Molecule** | **E-T1D MHC Class** | **E-T1D Antigen Name** |
| --- | --- | --- | --- | --- | --- | --- | --- | --- | --- | --- |
|  |  |  |  |  |  |  |  |  |  |  |
| 1 | ALIDV | AMVALIDVFHQ | ALYDV | EKMALYDVVSK | 65 | 41 | 0.31 | HLA-A*02:01 | MHC-I | Protein S100-B |
| 2 | ALIDVFH | AMVALIDVFHQ | ALSGVFC | ALSGVFCGV | 46 | 49 | no match | HLA-A*02:01 | MHC-I | Protein S100-B |
| 3 | LYHVYEV | ASLYHVYEVNL | LYVLFEV | TLLYVLFEVFD | 75 | 28 | 0.34 | HLA-A*02:01 | MHC-I | Insulin |
| 4 | SHLVEAL | CGSHLVEALYL | ADLVYAL | TMADLVYALRH | 58 | 46 | 0.22 | HLA-A*02:01 | MHC-I | Insulin |
| 5 | LTAVA | EFALTAVAEEV | WTAVA | MLLWTAVAVAV | 87 | 5 | 0.45 | HLA-A*02:01 | MHC-I | Potassium channel subfamily K member 16 |
| 6 | LTAVAE | FALTAVAEEV | LTLFAE | KVLTLFAEVE | 70 | 36 | 0.73 | HLA-A*02:01 | MHC-I | Insulin |
| 7 | LQANPVE | GGLQANPVEVQ | LQFIPVE | KALQFIPVESL | 78 | 24 | no match | HLA-A*02:01 | MHC-I | Zinc transporter 8 |
| 8 | LLPLLALL | GLLPLLALLA | WLPTGTLL | QWLPTGTLLV | 85 | 9 | 0.25 | HLA-A*02:01 | MHC-I | Zinc transporter 8 |
| 9 | QANPVEV | GLQANPVEVQS | DPNPPEV | IMDPNPPEVVS | 81 | 17 | no match | HLA-A*02:01 | MHC-I | Zinc transporter 8 |
| 10 | HLVEAL | GSHLVEALYL | DLVYAL | MADLVYALRH | 73 | 34 | 0.17 | HLA-A*02:01 | MHC-I | Protein S100-B |
| 11 | IVTDQK | GYIVTDQKPL | IVTDHR | SMIVTDHRYV | 7 | 52 | 0.65 | HLA-DRB3*02:02 | MHC-II | Receptor-type tyrosine-protein phosphatase-like N |
| 12 | GYIVTDQKPLS | GYIVTDQKPLS | GYIPLVGAPLG | GYIPLVGAPLG | 66 | 40 | 0.47 | HLA-A*02:01 | MHC-I | Insulin |
| 13 | LLIDLTSFL | HLLIDLTSFLL | LLYDANYFL | PLLYDANYFLC | 81 | 18 | no match | HLA-A*02:01 | MHC-I | Insulin |
| 14 | ILAVDGVLSV | ILAVDGVLSV | FLPVDFFPSV | FLPVDFFPSV | 83 | 14 | 0.85 | HLA-A*02:01 | MHC-I | Zinc transporter 8 |
| 15 | AVDGVLS | ILAVDGVLSVH | AVNGVLW | ATAVNGVLWTV | 78 | 23 | 0.14 | HLA-A*02:01 | MHC-I | Zinc transporter 8 |
| 16 | AVDGVLS | ILAVDGVLSVH | AVLEVLS | QPAVLEVLSAL | 63 | 43 | 0.62 | HLA-DRB3*02:02 | MHC-II | Receptor-type tyrosine-protein phosphatase-like N |
| 17 | IVTDQKPLSLA | IVTDQKPLSLA | ISFDQMERYLA | ISFDQMERYLA | 14 | 50 | no match | HLA-DRB3*02:02 | MHC-II | Receptor-type tyrosine-protein phosphatase-like N |
| 18 | IVTDQKPLSLAA | IVTDQKPLSLAA | ISFDQMERYLAA | ISFDQMERYLAA | 11 | 51 | 0.87 | HLA-DRB3*02:02 | MHC-II | Receptor-type tyrosine-protein phosphatase-like N |
| 19 | IVTDQKPLSLAA | IVTDQKPLSLAA | IVVNASASEAAA | IVVNASASEAAA | 56 | 48 | 0.47 | HLA-DRB3*02:02 | MHC-II | Receptor-type tyrosine-protein phosphatase-like N |
| 20 | IVTDQKPLSLAA | IVTDQKPLSLAA | IVSTAAQTFLAT | IVSTAAQTFLAT | 0 | 53 | no match | HLA-DRB3*02:02 | MHC-II | Receptor-type tyrosine-protein phosphatase-like N |
| 21 | KVNFFRMVISNP | KVNFFRMVISNP | NVNILVKQISTP | NVNILVKQISTP | 65 | 42 | 0.47 | HLA-DRB4*01:01 | MHC-II | Glutamate decarboxylase 2 |
| 22 | YLYNI | LAEYLYNIIKN | YLYNT | PALYLYNTGRS | 74 | 32 | 0.48 | HLA-A*02:01 | MHC-I | Insulin |
| 23 | ALWGPDPAA | LALWGPDPAAA | ALWALPHAA | EALWALPHAAA | 70 | 37 | 0.32 | HLA-A*02:01 | MHC-I | Insulin |
| 24 | PDPAAA | LALWGPDPAAA | PDTAAV | KLLPDTAAV | 77 | 26 | 0.3 | HLA-A*02:01 | MHC-I | Insulin |
| 25 | VDGVLSV | LAVDGVLSVHS | VDGVPFV | IFVDGVPFVVS | 59 | 45 | 0.52 | HLA-A*02:01 | MHC-I | Insulin |
| 26 | DVFHQY | LIDVFHQYSG | DVFHLY | YADVFHLYLQ | 58 | 47 | 0.55 | HLA-A*02:01 | MHC-I | Insulin |
| 27 | PLLALL | LLPLLALLAL | HLLVLL | LDHLLVLLEK | 74.3 | 30 | 0.44 | HLA-A*02:01 | MHC-I | Insulin |
| 28 | LLALL | LLPLLALLALW | LLGLL | GAFLLGLLFFV | 78.1 | 21 | 0.5 | HLA-A*02:01 | MHC-I | Zinc transporter 8 |
| 29 | LLALLA | LPLLALLALW | LLTLLA | LLLLTLLATV | 82.4 | 15 | 0.72 | HLA-A*02:01 | MHC-I | Insulin |
| 30 | LLALLAL | LPLLALLALWG | LLEWLAM | LLLEWLAMAV | 87.5 | 3 | 0.56 | HLA-A*02:01 | MHC-I | Insulin |
| 31 | LLALLAL | LPLLALLALWG | LLVTLAI | VFLLVTLAILT | 90.3 | 2 | 0.53 | HLA-A*02:01 | MHC-I | Insulin |
| 32 | LLALLAL | LPLLALLALWG | LLSTAAL | LTLLSTAALPV | 86.2 | 7 | 0.67 | HLA-A*02:01 | MHC-I | Insulin |
| 33 | MLMPVH | MLMPVHFLL | KLMPVC | KLMPVCVET | 86.7 | 6 | 0.75 | HLA-A*02:01 | MHC-I | Insulin |
| 34 | LLPLLA | MRLLPLLALL | LLTLLA | LLLLTLLATV | 75.8 | 27 | 1.1 | HLA-A*02:01 | MHC-I | Insulin |
| 35 | LLPLLAL | MRLLPLLALLA | LLEWLAM | LLLEWLAMAV | 62.9 | 44 | 1.13 | HLA-A*02:01 | MHC-I | Insulin gene enhancer protein ISL-1 |
| 36 | TRREAE | PKTRREAEDL | TRVEAQ | FLTRVEAQLH | 78.1 | 22 | 0.13 | HLA-A*02:01 | MHC-I | Receptor-type tyrosine-protein phosphatase-like N |
| 37 | RREAE | PKTRREAEDLQ | RRLAE | EARRRLAEMCF | 69.7 | 38 | 1.25 | HLA-A*02:01 | MHC-I | Receptor-type tyrosine-protein phosphatase-like N |
| 38 | LALLAL | PLLALLALWG | LAAYAL | KYLAAYALVG | 75 | 29 | 0.2 | HLA-A*02:01 | MHC-I | Receptor-type tyrosine-protein phosphatase-like N |
| 39 | ALLAL | PLLALLALWGP | ALLAF | REHALLAFTLG | 83.9 | 12 | 0.41 | HLA-A*02:01 | MHC-I | Receptor-type tyrosine-protein phosphatase-like N |
| 40 | ALLAL | PLLALLALWGP | ALWAL | TGEALWALPHA | 87.5 | 4 | 1.24 | HLA-A*02:01 | MHC-I | Urocortin-3 |
| 41 | LTAVAEEV | PLTAVAEEVE | LGAVQNEV | RLGAVQNEVI | 69.7 | 39 | 1.38 | HLA-A*02:01 | MHC-I | Insulin |
| 42 | PRLIAFTSEHSHFS | PRLIAFTSEHSHFS | ARLLSIRAMSTKFS | ARLLSIRAMSTKFS | 84.4 | 10 | 0.16 | HLA-A*02:01 | MHC-I | Insulin |
| 43 | RLLPLLALL | PRLLPLLALLE | RLNELLAYV | LRLNELLAYVS | 74.1 | 31 | 0.38 | HLA-DRB4*01:01 | MHC-II | Glutamate decarboxylase 2 |
| 44 | TNHFL | PVFTNHFLVEL | ANHFL | MNVANHFLSAP | 79.3 | 19 | 0.99 | HLA-A*02:01 | MHC-I | Insulin gene enhancer protein ISL-1 |
| 45 | AVDGVLS | RLAVDGVLSLL | AVNGVMW | GTAVNGVMWTV | 93.3 | 1 | 0.19 | HLA-A*02:01 | MHC-I | Insulin |
| 46 | LLPLLALL | RLLPLLALLA | LLVTLAIL | FLLVTLAILT | 73.5 | 33 | 1.39 | HLA-A*02:01 | MHC-I | Insulin |
| 47 | LLPLLALL | RLLPLLALLA | LLCLIFLL | LLLCLIFLLV | 85.3 | 8 | 0.59 | HLA-A*02:01 | MHC-I | Insulin |
| 48 | HLVEALYLV | SHLVEALYLVC | HLDDVGFLV | RHLDDVGFLVA | 73 | 35 | 0.49 | HLA-B*08:01 | MHC-I | Insulin |
| 49 | ILKDFSILL | TILKDFSILLM | ILSPFLPLL | SILSPFLPLLP | 84.4 | 11 | 0.24 | HLA-A*02:01 | MHC-I | Insulin |
| 50 | RLLPLL | WMRLLPLLAL | HLLVLL | LDHLLVLLEK | 77.4 | 25 | 0.3 | HLA-A*02:01 | MHC-I | Neuroendocrine convertase 2 |
| 51 | RLLPLL | WMRLLPLLAL | PFLPLL | LSPFLPLLPI | 82.4 | 16 | 0.18 | HLA-B*08:01 | MHC-I | Insulin |
| 52 | LLPLL | WMRLLPLLALL | LLGLL | GAFLLGLLFFV | 83.3 | 13 | 0.98 | HLA-A*02:01 | MHC-I | Zinc transporter 8 |
| 53 | ALLGIPLNV | YALLGIPLNVI | ALLGLTLGV | MALLGLTLGVL | 78.8 | 20 | 0.4 | HLA-A*02:01 | MHC-I | Glutamate decarboxylase 2 |

**Table S3:** HLA amino acid sequences used in this work.

**HLA-A*02:01**

MAVMAPRTLVLLLSGALALTQTWAGSHSMRYFFTSVSRPGRGEPRFIAVGYVDDTQFVRFDSDAASQRMEPRAPWIEQEGPEYWDGETRKVKAHSQTHRVDLGTLRGYYNQSEAGSHTVQRMYGCDVGSDWRFLRGYHQYAYDGKDYIALKEDLRSWTAADMAAQTTKHKWEAAHVAEQLRAYLEGTCVEWLRRYLENGKETLQRTDAPKTHMTHHAVSDHEATLRCWALSFYPAEITLTWQRDGEDQTQDTELVETRPAGDGTFQKWAAVVVPSGQEQRYTCHVQHEGLPKPLTLRWEPSSQPTIPIVGIIAGLVLFGAVITGAVVAAVMWRRKSSDRKGGSYSQAASSDSAQGSDVSLTACKV

**HLA-B*08:01**

MLVMAPRTVLLLLSAALALTETWAGSHSMRYFDTAMSRPGRGEPRFISVGYVDDTQFVRFDSDAASPREEPRAPWIEQEGPEYWDRNTQIFKTNTQTDRESLRNLRGYYNQSEAGSHTLQSMYGCDVGPDGRLLRGHNQYAYDGKDYIALNEDLRSWTAADTAAQITQRKWEAARVAEQDRAYLEGTCVEWLRRYLENGKDTLERADPPKTHVTHHPISDHEATLRCWALGFYPAEITLTWQRDGEDQTQDTELVETRPAGDRTFQKWAAVVVPSGEEQRYTCHVQHEGLPKPLTLRWEPSSQSTVPIVGIVAGLAVLAVVVIGAVVAAVMCRRKSSGGKGGSYSQAACSDSAQGSDVSLTA

**HLA-DRB3*02:02**

MVCLKLPGGSSLAALTVTLMVLSSRLAFAGDTRPRFLELLKSECHFFNGTERVRFLERHFHNQEEYARFDSDVGEYRAVRELGRPDAEYWNSQKDLLEQKRGQVDNYCRHNYGVGESFTVQRRVHPQVTVYPAKTQPLQHHNLLVCSVSGFYPGSIEVRWFRNGQEEKAGVVSTGLIQNGDWTFQTLVMLETVPRSGEVYTCQVEHPSVTSPLTVEWSARSESAQSKMLSGVGGFVLGLLFLGAGLFIYFRNQKGHSGLQPTGFLS

**HLA-DRB4*01:01**

MVCLKLPGGSCMAALTVTLTVLSSPLALAGDTQPRFLEQAKCECHFLNGTERVWNLIRYIYNQEEYARYNSDLGEYQAVTELGRPDAEYWNSQKDLLERRRAEVDTYCRYNYGVVESFTVQRRVQPKVTVYPSKTQPLQHHNLLVCSVNGFYPGSIEVRWFRNSQEEKAGVVSTGLIQNGDWTFQTLVMLETVPRSGEVYTCQVEHPSMMSPLTVQWSARSESAQSKMLSGVGGFVLGLLFLGTGLFIYFRNQKGHSGLQPTGLLS

**Figure S1**: Comparison of the structures if the T1DM epitopes (in green) and Infectious Epitopes (in red) bided to the corresponding HLA molecule (in grey). Pair numbers as indicated in Table S2.

**Pair 1:** A contact overlap of about 65% indicates that the two epitope peptides occupy largely the same binding groove region and engage many of the same HLA residues.

**Pair 2:** The epitope peptides bind the same HLA groove and share the core anchoring region, but their detailed binding footprints differ substantially, overlap 46%. Their binding modes are only partially conserved.

**Pair 3:** Shows a 75% overlap indicating that the epitope peptides engage nearly the same HLA binding groove residues and likely adopt a broadly similar binding register.

**Pair 4:** The two epitopes share a substantial portion of the HLA-binding footprint. Structurally, this suggests they occupy a broadly similar region of the epitope peptide-binding groove but differ in several local interactions. Moderate HLA-binding similarity (~58%).

**Pair 5:** Very high HLA-binding similarity (~87%). The two epitope peptides engage almost exactly the same set of HLA groove residues.

**Pair 6**: High HLA-binding similarity (~70%). The two epitope peptides engage most of the same residues in the HLA groove and likely occupy a very similar binding register.

**Pair 7**: High HLA-binding similarity (~78%). The two epitope peptides engage nearly the same set of HLA groove residues and likely occupy a very similar binding register.

**Pair 8:** Very high HLA-binding similarity (~85%). The epitope peptides engage nearly identical sets of HLA groove residues, suggesting a highly conserved binding mode.

**Pair 9:** Very high HLA-binding similarity (~81%). The epitope peptides engage almost exactly the same HLA groove residues and likely adopt a very similar binding register.

**Pair 10:** High HLA-binding similarity (~73%). The epitope peptides engage most of the same HLA groove residues and are likely to adopt a similar binding register.

**Pair 11:** Very low HLA-binding similarity (~7%). Although both epitope peptides are 11-mers and share the initial GYI motif, their interaction footprints with the HLA molecule are largely distinct.

**Pair 12:** Moderately high HLA-binding similarity (~66%). The epitope peptides engage many of the same residues within the HLA groove, suggesting a comparable binding register.

**Pair 13:** Very high HLA-binding similarity (~81%). Despite sequence differences, the epitope peptides engage nearly identical sets of HLA groove residues, suggesting a highly conserved binding footprint and a similar binding register.

**Pair 14:** Very high HLA-binding similarity (~83%). The two epitope peptides interact with almost the same set of HLA groove residues, indicating a highly conserved binding footprint and likely a very similar binding register.

**Pair 15:** High HLA-binding similarity (~78%). Based on HLA-contact similarity, it is a plausible structural mimicry candidate, although it is not quite as strong as the top-performing pairs.

**Pair 16:** Moderately high HLA-binding similarity (~63%). It shows evidence of a conserved binding register, but the footprint similarity is not as strong as the leading candidates.

**Pair 17:** Low HLA-binding similarity (~14%). Based on the contact footprint alone, Pair 17 is not a strong structural mimicry candidate and ranks near the bottom of the set analyzed so far.

**Pair 18:** Very low HLA-binding similarity (~11%). Based on HLA-contact footprint similarity, Pair 18 is not a strong structural mimicry candidate and falls into the same low-similarity group.

**Pair 19:** Moderate HLA-binding similarity (~56%). Unlike the strongest pairs (>80% overlap), these epitope peptides share only about half of their HLA-contact footprint. They likely occupy the same general groove but make noticeably different local interactions.

**Pair 20:** In fact, the two models appear to use completely different sets of HLA residues for epitope peptide contacts. Overlap 0%**.**

**Pair 21:** Moderately high HLA-binding similarity (~65%). It is not among the strongest mimicry candidates, but it shows a reasonably conserved HLA-binding footprint and would be considered a plausible, though not top-tier, structural similarity candidate.

**Pair 22:** High HLA-binding similarity (~74%). It shows a substantially conserved HLA-binding footprint and would be considered a plausible structural mimicry candidate based on HLA contacts alone. The overlap (~74%) places Pair 22 in the same range as several of the stronger candidates.

**Pair 23:** This pair shows high HLA-binding similarity. The overlap (~70%) indicates that the epitope peptides appear to use a very similar set of HLA groove residues and likely adopt a comparable binding register.

**Pair 24:** High HLA-binding similarity (~77%). Pair 24 belongs in the upper tier of candidates and is very similar to Pairs 7 and 15. Based on HLA-contact footprint similarity, it is a strong candidate for similar HLA presentations and potential structural mimicry.

**Pair 25:** Moderate HLA-binding similarity (~59%). Pair 25 falls into the middle tier, close to Pairs 4 and 19. It shows evidence of a conserved binding footprint but is not among the strongest structural mimicry candidates.

**Pair 26:** Moderate HLA-binding similarity (~58%). Pair 26 falls into the middle tier, like Pairs 4 and 25. It shows a conserved core binding footprint but is not among the strongest structural mimicry candidates. While the epitope peptides are compositionally similar and engage many of the same groove residues, the overlap is substantially lower than the strongest candidates (>80%). Differences in side-chain placement appear to alter part of the HLA interaction footprint.

**Pair 27**: High HLA-binding similarity. Pair 27 appears to be another strong candidate for similar HLA presentation and potential molecular mimicry, with an overlap of 74%.

**Pair 28:** High HLA-binding similarity (78.1%) Pair 28 falls into the same high-similarity group as Pairs 7 and 15.

**Pair 29**: Very high HLA-binding similarity (82.4%) Pair 29 is within the top tier of pairs. The epitope peptides engage nearly identical HLA groove residues, differing at only a handful of positions.

**Pair 30:** Extremely high HLA-binding similarity (87.5%). The two epitope peptides contact almost exactly the same set of HLA groove residues, differing at only four positions across the entire contact footprint**.**

**Pair 31**: Extremely high HLA-binding similarity (90.3%). Pair 31 is at the top of the priority list alongside Pairs 30, 5, 8, 14, and 29.

**Pair 32:** Very high HLA-binding similarity (86.2%) Pair 32 belongs among the strongest matches in the entire set. The two epitope peptides engage almost exactly the same HLA groove residues, differing at only a few contact positions.

**Pair 33:** Very high HLA-binding similarity (86.7%) Pair 33 is one of the strongest matches in the entire set. The two epitope peptides differ by only four contact residues across the whole HLA-binding footprint.

**Pair 34**: High HLA-binding similarity (75.8%). Pair 34 falls into the same high-similarity tier as Pairs 3, 22, 24, 27, and 28. The epitope peptides engage most of the same HLA groove residues, although not quite as extensively as the strongest candidates (Pairs 31, 30, 5, 33, and 32).

**Pair 35**: Moderately high HLA-binding similarity (62.9%) Pair 35 shows a conserved core HLA-binding footprint, but it is clearly less similar than the top-ranked pairs (>80%). The two epitope peptides share 22 HLA-contact residues, while maintaining several distinct contacts on each side.

**Pair 36:** High HLA-binding similarity (78.1%) Despite the epitope peptides having quite different sequences, they engage nearly the same HLA groove residues. Only seven contact residues differ between the two footprints.

**Pair 37**: High HLA-binding similarity (69.7%). Pair 37 sits right around the boundary between the moderate-high and high-similarity groups. The epitope peptides share most of the core HLA groove contacts, but each also makes several unique interactions**.**

**Pair 38:** High HLA-binding similarity (75.0%). Pair 38 falls into the same range as Pair 3 (75%) and just below Pair 34 (75.8%). The epitope peptides share most of the core HLA groove contacts, with only a handful of differences.

**Pair 39:** Very high HLA-binding similarity (83.9%). Pair 39 is one of the strongest matches in the entire dataset. The two epitope peptides share 26 of 31 unique HLA-contact residues and differ at only five contact positions.

**Pair 40:** Extremely high HLA-binding similarity (87.5%). Pair 40 is one of the strongest matches in the entire dataset. The two epitope peptides differ at only four contact residues across the complete HLA-contact footprint.

**Pair 41:** High HLA-binding similarity (69.7%). Pair 41 shows a conserved core HLA-binding footprint but does not reach the top tier of the dataset. Its overlap is essentially identical to Pair 37 (69.7%).

**Pair 42:** Very high HLA-binding similarity (84.4%). Pair 42 is one of the strongest matches in the entire dataset. The two epitope peptides share 27 of 32 total HLA-contact residues and differ at only five positions.

**Pair 43:** High HLA-binding similarity (74.1%). Pair 43 falls into the same range as Pairs 22 (74.0%), 27 (74.3%), and slightly above Pair 10 (73.0%).

**Pair 44:** High HLA-binding similarity (79.3%). Pair 44 is one of the stronger matches in the dataset. The epitope peptides share 23 of 29 unique HLA-contact residues and differ at only six contact positions.

**Pair 45:** Extremely high HLA-binding similarity (93.3%). The two epitope peptides use essentially the same HLA-binding footprint, differing at only two contact residues.

**Pair 46**: High HLA-binding similarity (73.5%). Pair 46 falls into the same range as Pairs 10 (73.0%), 22 (74.0%), and 27 (74.3%). The epitope peptides share a substantial fraction of their HLA-binding footprint, but they are not among the top >80% matches.

**Pair 47:** Very high HLA-binding similarity (85.3%). Pair 47 is one of the strongest matches in the entire dataset. The two epitope peptides share 29 of 34 total HLA-contact residues and differ at only five contact positions.

**Pair 48:** High HLA-binding similarity (73.0%). Pair 48 falls into the same cluster as Pair 10 (~73.0%) and just below Pairs 22 (~74.0%) and 27 (~74.3%). The epitope peptides share a large core of HLA groove contacts but have several distinct interactions on each side.

**Pair 49:** Very high HLA-binding similarity (84.4%). Pair 49 is one of the strongest matches in the dataset. The two epitope peptides share 27 of 32 total HLA-contact residues and differ at only five contact positions.

**Pair 50:** High HLA-binding similarity (77.4%). Pair 50 falls into the same cluster as Pairs 7 (78.0%), 15 (78.0%), 28 (78.1%), and 36 (78.1%). The two epitope peptides share most of their HLA-binding footprint and differ at only a handful of contact residues.

**Pair 51:** Very high HLA-binding similarity (82.4%). Pair 51 belongs to the top tier of candidates. The two epitope peptides share 28 of 34 total HLA-contact residues and differ at only a handful of positions.

**Pair 52**: Very high HLA-binding similarity (83.3%). Pair 52 is one of the strongest matches in the entire dataset. The two epitope peptides share 25 of 30 total HLA-contact residues and differ at only a few positions.

**Pair 53:** High HLA-binding similarity (78.8%). Pair 53 joins the upper tier of strong matches. The two epitope peptides share 26 of 33 total HLA-contact residues and differ at only a small number of positions.

**Figure S2.** Enlarged epitopes from Pair 45 bound to the corresponding HLA molecules showing that the superposition is almost perfect in the original epitopes binding region.
